# Editorial

**Published:** 1855-07

**Authors:** 


### Editorial.

---

### THE GREAT DENTAL PATENT CASE OF DR. JOHN ALLEN *vs* WM. M. HUNTER.

This case which has so long kept the Profession in a state of suspense, is now decided, and we have been at much trouble and expense to procure a fair and impartial report for the Profession. To all engaged in continous gum work or those who wish to be posted in the Profession, the report will be of great interest. It embodies a vast amount of scientific matter in relation to the material used and a complete history of the improvement itself, showing the true reason for the failure of Delabarre's formula, and the success of Allen and Hunter's. We think a careful perusal of the report will explain the seeming contradiction, of much of the testimony, and the reason why so many have failed. That the *thing* itself is "worthy the attention of the Profession," we think there can now be no doubt. Whatever may be the opinion of many in the Profession as to its practical use, we feel assured that no one doing much in mechanical Dentistry will be able to get along in all cases satisfactorily without it.

In connection with the report we have published the cards of Drs. Hunter and Allen, and have no doubt but the profession will generally be glad to know that these gentlemen have buried the *hatchet* and have smoked together the *calumet* of peace.

We have quite a lengthy report from Dr. J. M. Locke which was introduced as testimony in the case, and which we intended giving in full instead of the abstract we have given, but it was received too late for insertion in its proper place. As a matter of scientific interest we shall give this in the next number of the Register.

Our regular subscribers who have been paying for the Register will get the report as a matter of just due, and will we hope indulge us for the delay in the July number, of the Register. This has been unavoidable. There are many who have for some time been taking the Regsiter, who will be supplied with the present number whenever they make their remittance of dues.

The report will be for sale at Messrs Jones, White & McCurdy, N. Y.; Sutter & Rayner, do; New-York Teeth Manufacturing Company, do; and J. D. Chevalier's, do; Messrs Jones, White & McCurdy, Boston; and Codman's Dental Depot, do; Messrs Jones, White & McCurdy, Philadelphia; and Oram & Armstrong do; Francis Arnolds Baltimore, Md.; J.B. Dunlevy Pittsburgh, Pa.; Hyde Goodrich, New Orleans; T. L. Rives, St. Louis; J. T. Hughes, Louisville; J. M. Brown's Cincinnati; or by application to the editor of the Dental Register. Price single copies 75 cents, three copies, $2,00.

---

DENTAL REGISTER.

It will be seen by the prospectus on the second page of our cover that the terms of the Register hereafter will be three dollars per annum. The present number has been enlarged a few pages over the size of our future plans. This was necessary to enable us to publish in full the report of the trial of Drs. Allen and Hunter.

Our design is to make the Register such a periodical as the profession will not willingly be without; but to do this it will take more of our time and much more of our means than we have heretofore been giving. It rests with the profession whether in the latter we shall be reimbursed. We are encouraged from almost every quarter to expect an enlarged circulation. We hope this will be the case—yet we wish this one thing perfectly understood that those who receive the Register regularly must expect to pay for it. In this affair above all others we wish to be independent, for we intend that the Register shall be perfectly independent. We feel perhaps as willing as any man in the profession to give our knowledge and even some of our time for nothing, but we do not feel that we are at liberty to give thus freely the productions of some of our best writers and the labor of the type setters, paper makers, etc., etc.

In all seriousness therefore, we shall expect those who take the Register to pay for it, and wish it distinctly understood that when taken time after time from the post office, the law makes such liable, those therefore who receive more than one number of the Register unless they wish to be considered a subscriber should remail it "Dental Register Cincinnati, O." and plainly write their name and post office on the wrapper or on the cover of the work itself. We some times receive the Register returned without any possible means of knowing from whence it comes, such notice as a matter of course is invalid, and cannot be taken notice of.

We write plain because we wish to be understood, and give no occasion for hard thoughts hereafter. To the many who have thus far assisted us in their contributions, and with their regular dues we return out heartfelt thanks and hope to continue our quarterly visits to them. All good current money will be taken in payment, and when remitted by mail and letter registered it will be at our risk. Five dollars in advance will be taken for two years subscription.

### SELF INJECTING EAR SYRINGE.

We acknowledge the receipt of one of these very neat yet simple and valuable instruments, invented by Dr. S. P. Hullihen, of Wheeling, Va., who not content with the regular routine of dental practice and improvement, is continually making himself known as a general surgeon.

This instrument possesses two very excellent advantages over the old methods of injecting the ear. First the patient can use the instrument; and second, the cup attached to the instrument itself catches the overflowing from the ear.

They are for sale by Henry E. Blair, south west corner of eighth and Walnut streets Philadelphia, Pa., and by his appointed agents.

### DENTAL OBTURATER.

This new Dental Periodical which we spoke of in our last issue is now before us, and is gotten up with much spirit and presents a well selected sheet of instructive matter for the reader. We wish the Obturater and its indefatigable editor success.

